# New epidemiological aspects of visceral and cutaneous leishmaniasis in Taza, Morocco

**DOI:** 10.1186/s13071-016-1910-x

**Published:** 2016-11-29

**Authors:** Maryam Hakkour, Asmae Hmamouch, Mohamed Mahmoud El Alem, Abdelkbir Rhalem, Fatima Amarir, Mohamed Touzani, Abderrahim Sadak, Hajiba Fellah, Faiza Sebti

**Affiliations:** 1 National Reference Laboratory of Leishmaniasis, National Institute of Hygiene, Agdal, Rabat, Morocco; 2 Laboratory of Zoology and General Biology, Faculty of Science, Agdal, Rabat, Morocco; 3 Hassan II Institute of Agronomy and Veterinary, Rabat, Morocco; 4 Laboratory of Microbial Biotechnology, Faculty of Sciences and Techniques, Sidi Mohammed Ben Abdellah University, Fez, Morocco; 5 Institute of Nursing Professions and Health Techniques, Casablanca, Morocco; 6 Delegation of Health, Taza, Morocco

**Keywords:** Leishmaniasis, *Leishmania infantum*, *Leishmania tropica*, ITS1-PCR-RFLP, Taza Province, Morocco

## Abstract

**Background:**

Leishmaniasis is considered among the main endemic diseases in Morocco. However, further knowledge about epidemiological aspects of this disease is needed in several provinces to plan control and preventive strategies to tackle the disease. The present study aims to determine the epidemiological aspect of cutaneous and visceral leishmaniasis in Taza Province from 2007–2014 and to identify the circulating species in this province.

**Results:**

The temporal study from 2007 to 2014 showed that the number of cutaneous leishmaniasis cases increased since 2010. During the period of study, most leishmaniasis cases were detected in both urban and rural areas with 34% of cases detected in two urban localities, Bab Zitouna and Bab tété with 297 and 106 cases, respectively. The molecular study of cutaneous leishmaniasis showed the presence of non-sporadic *Leishmania infantum* and *Leishmania tropica* in this province. Regarding visceral leishmaniasis, *Leishmania infantum* is the species that has been identified.

**Conclusions:**

The epidemio-molecular study of leishmaniasis in Taza Province showed the coexistence of two species of *Leishmania* in the same foci. They also indicated that CL due to *Leishmania infantum* is more prevalent than reported in the literature. These results will be helpful for the implementation of control strategies by targeting dogs that constitute a reservoir of *Leishmania infantum*.

**Electronic supplementary material:**

The online version of this article (doi:10.1186/s13071-016-1910-x) contains supplementary material, which is available to authorized users.

## Background

Leishmaniasis is a parasitic protozoan disease caused by species of the genus *Leishmania*, and one of the neglected tropical diseases that has been among the major causes of morbidity and mortality. In addition, it is known to be the ninth largest disease burden among the 13 parasitic and bacterial neglected tropical diseases worldwide. In Morocco, leishmaniasis is an endemic disease which has both visceral and cutaneous forms reported [1].

The visceral leishmaniasis (VL) caused by *Leishmania infantum* Mon-1, has been endemic in Morocco for several decades, with 152 cases reported per year. The first case of infantile kala-azar was suspected in Tanger in 1921 [2] where it was essentially limited to northern Morocco but has now shown a remarkable sporadic extension over the whole territory [3].

Cutaneous leishmaniasis (CL) is caused by three clinically important *Leishmania* species (*Leishmania major*, *Leishmania tropica* and *Leishmania infantum*) [4]. The zoonotic CL caused by *Leishmania major* zymodeme MON-25 has been known since 1914 [5] and it manifests as endemic and epidemic in the pre-Saharan areas [6]. Thereafter, this disease is presented as epidemics alternated by lull periods. Cutaneous leishmaniasis caused by *L. infantum* was reported for the first time in Morocco in 1996 at the Central Rif in Taounate [7], then in 2007 in other parts of northern Morocco, particularly in the Province of Sidi Kacem [4] and in 2014 in Sefrou Province [8]. Cutaneous leishmaniasis caused by *Leishmania tropica* has the largest geographical distribution in Morocco compared to Maghreb countries. The transmission cycle of this species in Morocco remains poorly known. The presence of *L. tropica* (MON-279) in a dog with symptoms of canine VL [9] suggest that the cycle is zoonotic. Nevertheless, the small number of canine cases and the short duration of the lesions make it difficult to define the precise role of the dog in the epidemiological cycle [10]. Therefore, the transmission cycle was also suggested to be anthroponotic [11].

The first case of CL due to this species was identified in 1987 in France from a Moroccan child who stayed at Tanant [12]. Thereafter, widespread sparse hypoendemic rural foci were identified in a sub-arid area ranging from Tadla to Agadir. Since then, large outbreaks have appeared, extending from Agadir-Guelmim in the South via Essaouira and Chichaoua in the West, Beni Mellal, Azilal, Marrakech in the Centre, Ouarzazate in the East to Taza in the North [11].

The epidemiological situation of leishmaniasis remains worrying despite efforts to control this disease. Although the number of cases due to *L. major* have decreased significantly, the number of cases due to *L. tropica* is still high and stable with an average of 1550 cases reported between 2000 and 2014 [13].

In 2014, CL due to *L. tropica* was recorded in 37 provinces according to clinical diagnosis and is still increasing in eight provinces considered as foci for leishmaniasis, including Taza Province. This province is situated beside endemic foci of CL. The numbers of CL cases have continued to increase since 2010. In addition, the presence of VL in this province presents several questions about the epidemiological characteristics of this province and the causative parasite species.

This study aims to determine the epidemiological data of leishmaniasis in Taza Province and to identify the *Leishmania* parasite responsible for the recent cases of CL and VL leishmaniasis.

## Methods

### Study area

**Fig. 1:**
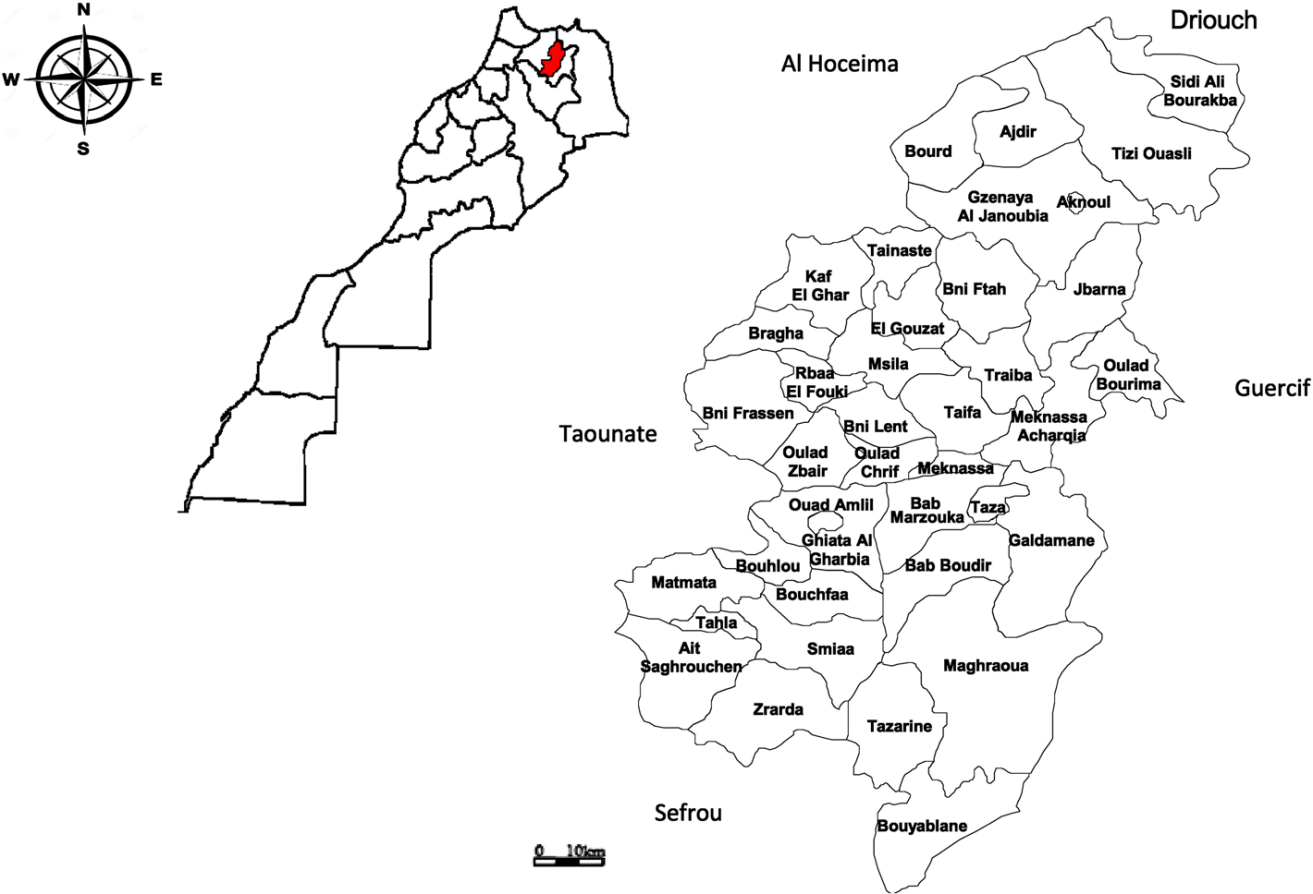
Taza Province on the map and its districts [14]

Taza Province is located in north-eastern Morocco and extends over an area of 7098.50 km^2^ (Fig. 1). It is bounded to the north by Al Hoceima and Driouch provinces, to the west by Taounate Province to the south by Sefrou Province and to the east by Guercif Province. Taza Province is characterized by a Mediterranean climate: cold-wet winter and semi-arid summer. The temperature varies between 1.4 °C and 45.2 °C and the average rainfall is between 100 and 200 mm/year in arid areas and exceeds 500 mm/year in the wetland areas [14].

### Sampling

Data for patients with clinical lesions corresponding to CL were collected during 2000–2014 from the Provincial Service of Infrastructure and Ambulatory Actions of Taza Province. Patients with clinical lesions of CL were examined for *Leishmania* by smear microscopy, at the Provincial Laboratory and slides were sent to the National Reference Laboratory of Leishmaniasis at the National Institute of Hygiene of Morocco (NIH) in Rabat for confirmation. Epidemiological data concerning patients (including age, sex and location) and lesions (morphological appearance, siege, size and number) were kindly provided for each patient.

### Molecular analysis

In 2014, 227 cases of CL and 6 of VL were declared, only 66 (63 CL and 3 VL) were examined by molecular study, the remaining cases were not received in the National Laboratory of Leishmaniasis in the National Institute of Hygiene (NIH Morocco) and others were defective. The extraction was done using the Blood and Tissue kit (Qiagen, Hilden, Germany), following the manufacturer’s instructions. The internal transcribed spacer (ITS1) of the ribosomal DNA was amplified using the primer pair L58S and LITSR [15, 16]. PCR products were digested with the restriction endonuclease Mn1-I. Reference strains of *L. tropica* (MHOM/AZ/1974/SAF-K27), *L. major* (MHOM/IL/1967/Jericho) and *L. infantum* (MHOM/TN/1980/IPT1) were used as positive controls.

### Statistics

Statistical analysis was performed by the software R. Pearson’s Chi-square test (*χ* ^*2*^) was used for comparison of age and sex. For all tests, the significance level was 0.05. Statistical analysis of sectorial distribution of cases was used by Fisher’s exact test. According to this test, the dependent variable was the total number of cases and the explanatory variables were the locality and the year of sampling.

## Results

### Temporal and spatial distribution of VL and CL in Taza Province

**Fig. 2:**
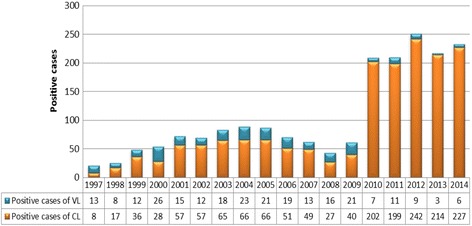
VL and CL cases between 1997 and 2014 in Taza Province

Taza Province was known to be a hypoendemic focus of VL (caused by *L. infantum*). Between 1991 and 1995, 23 cases of VL were recorded [17]. Between 1997 and 2014, the Moroccan Ministry of Health reported 253 human VL cases in this focus, the fluctuation of VL cases was noted with an average of 14 cases per year (Fig. 2). The incidence rate remained relatively stable between 0.53 and 4.11 with an average of 2.27.

From 1997 to 2014, 1651 cases of CL were noted and distributed into two phases. First, a hypoendemic phase from 1997 to 2009 with only 567 cases and an average of 44 cases per year. Secondly, a highly endemic phase was recorded from 2010 to 2014 with a significant increase (1084 cases) and an average of 216 cases per year (Fig. 2). The incidence of CL has recorded a significant increase (Pearson Chi-square test: *χ* ^2^ = 242.9134, *df* = 17, *P* < 0.0001) which jumped from 6.95/100,000 inhabitants between 1997–2009 to 38.81/100,000 inhabitants between 2010–2014.

### Spatial distribution

**Fig. 3:**
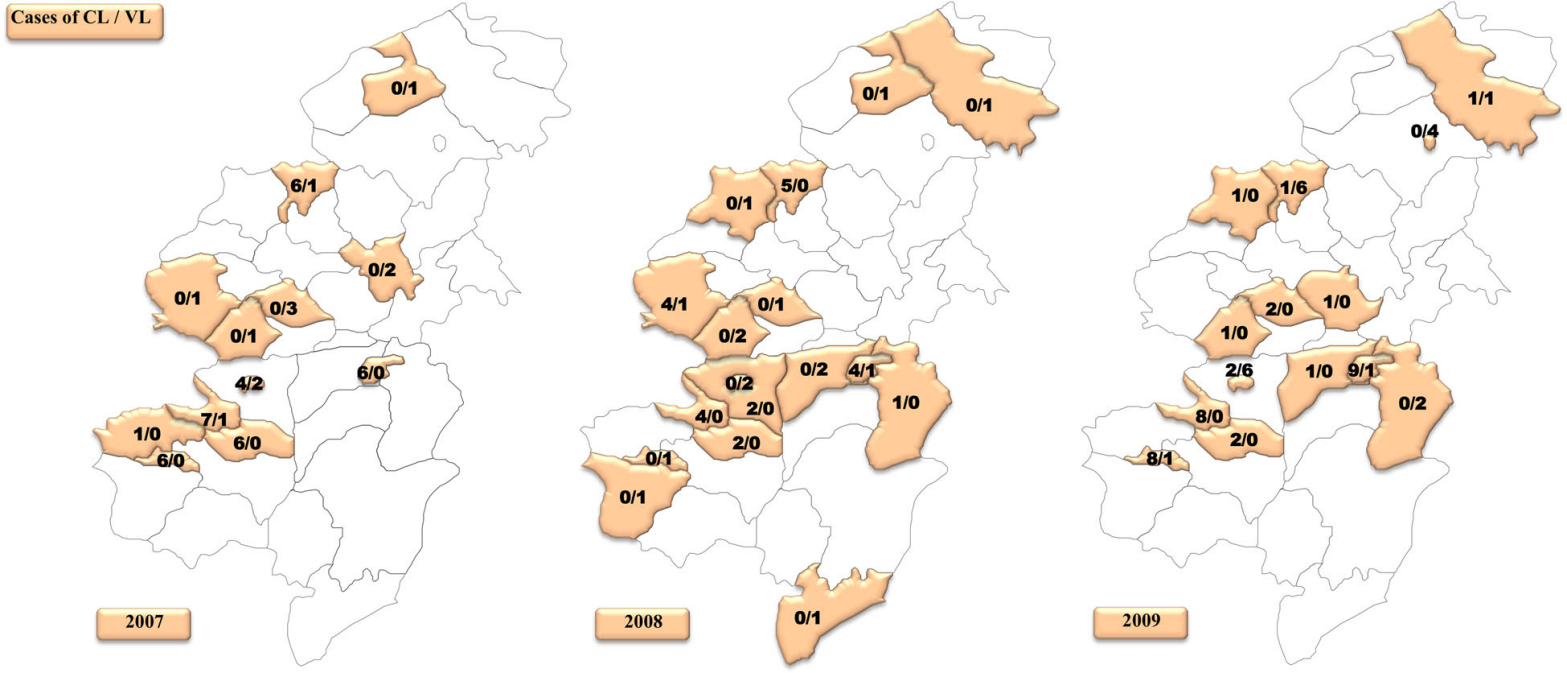
Geographical distribution of VL and CL cases in Taza Province (2007–2009)

**Fig. 4:**
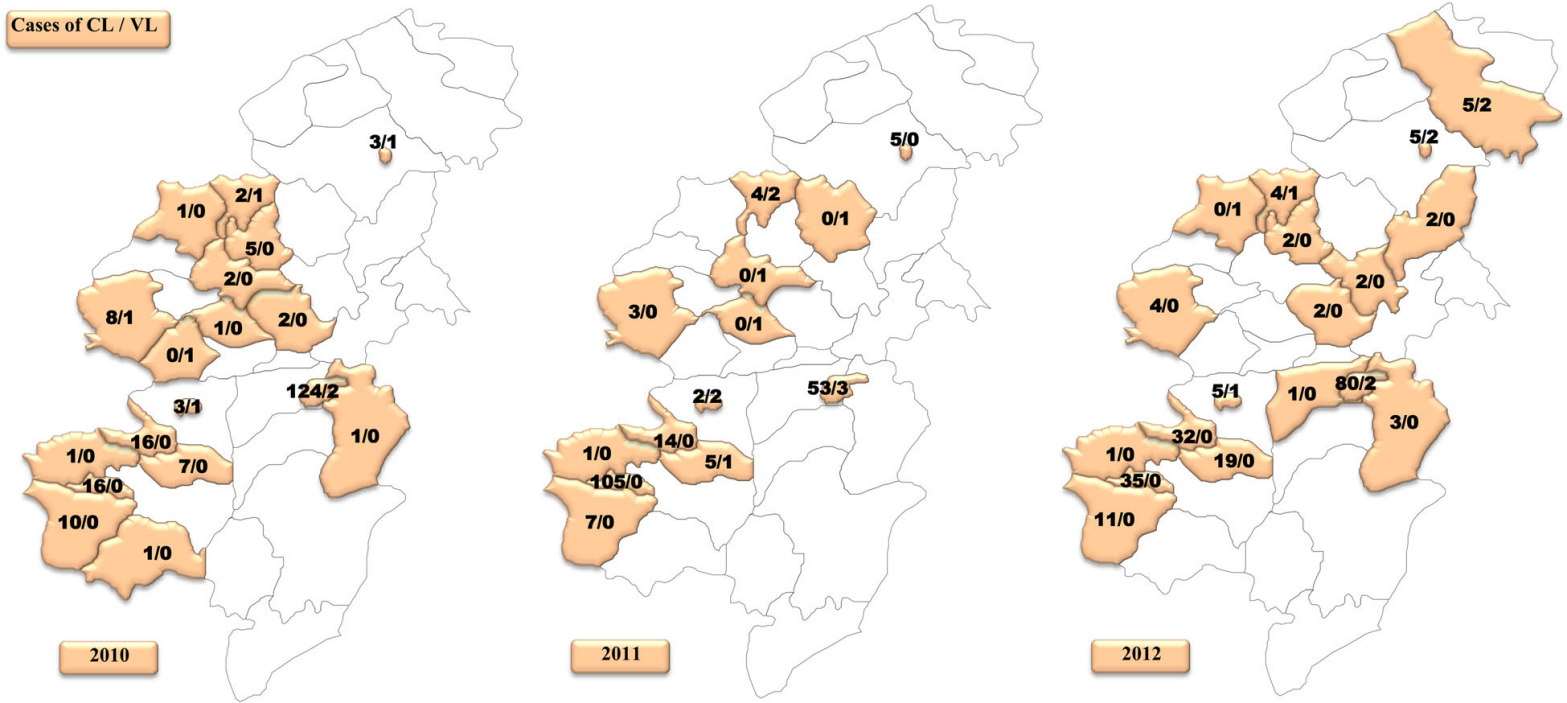
Geographical distribution of VL and CL cases in Taza Province (2010–2012)

**Fig. 5:**
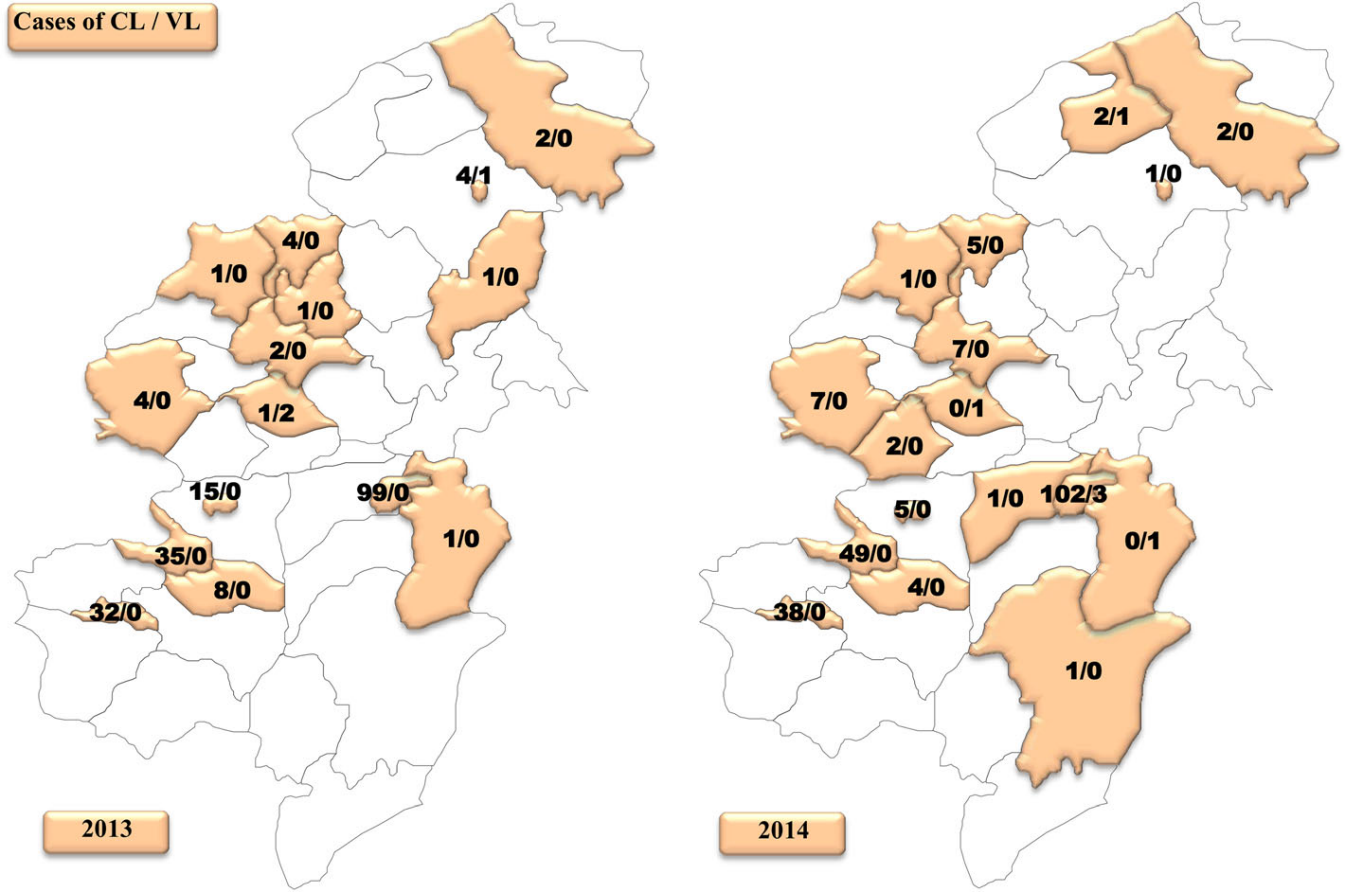
Geographical distribution of VL and CL cases in Taza Province (2013–2014)

Leishmaniasis affected both urban (58%) and rural (42%) areas in Taza Province. The geographical study (Figs. 3, 4, 5) showed that a total of 22 among 39 sectors were affected by VL in Taza Province between 2007 and 2014. However, in 2007, 8 sectors were affected by VL. This number decreased to 6 sectors in 2012 and to 4 sectors in 2014. This study also showed that the rural sectors of Taza Province lead with 87% of VL cases. In fact, most of the reported cases were from Taza high, Beni Lent, Ouad Amlil, Aknoul, Tainaste, Kaf El Ghar, Ajdir, Oulad Zbair and Bni Frassen. The sporadic cases appeared in other sectors.

With reference to CL, 24 sectors were recorded from 2007 to 2014. Seven sectors were affected with a total of 49 cases in 2007. In 2012, the number of positive cases of CL jumped to 242 cases across 17 sectors. In 2014, 227 cases were distributed among 15 sectors (Figs. 3, 4, 5).

**Fig. 6:**
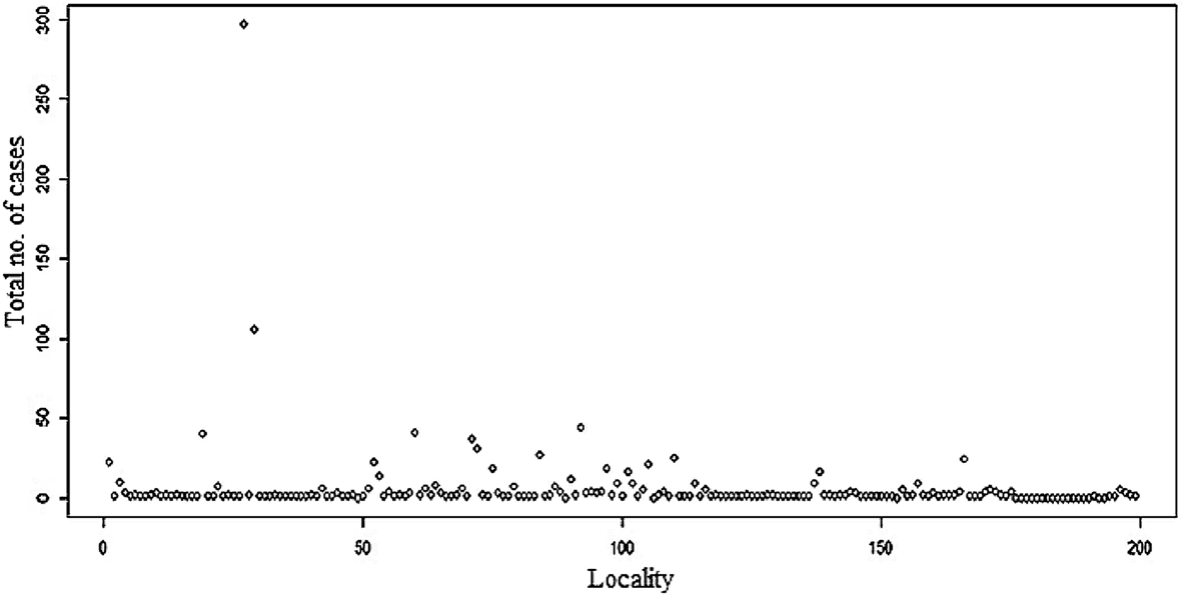
Distribution of leishmaniasis cases in Taza’s localities (2007–2014)

**Fig. 7:**
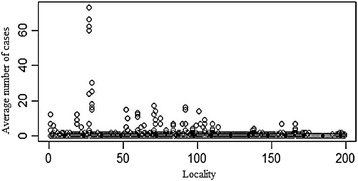
Average number of leishmaniasis cases in Taza’s localities (2007–2014)

Figures 6 and 7 show a plot distribution of leishmaniasis cases reported between 2007 and 2014 in different localities (a total of 199) (Additional file 1: Table S1) of Taza Province. The area could be grouped into three layers of distribution: (i) a group where the numbers of leishmaniasis cases is 297 (observed in Bab Zitouna locality); (ii) a group where the numbers of leishmaniasis cases is more than 100 cases (observed in Bab tété locality); (iii) a group including the remaining localities (197) where the number of cases was less than 50.

**Fig. 8:**
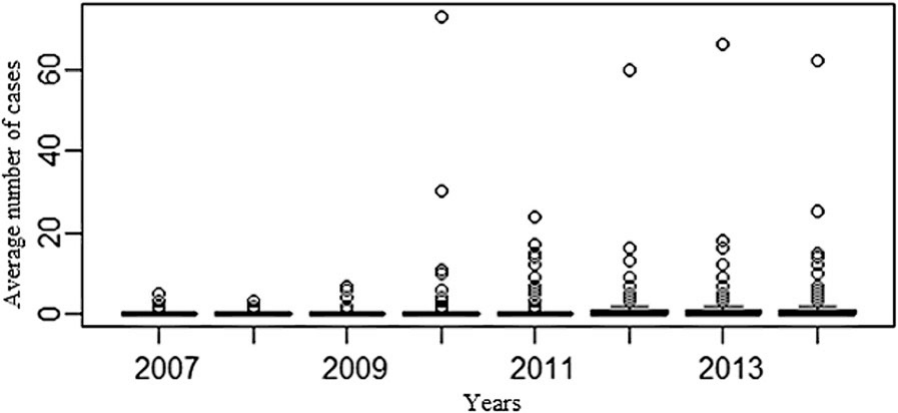
Variation of leishmaniasis cases in Taza’s localities in relation to year (2007–2014)

The plot of the distribution of VL and CL cases depending on year (Fig. 8) shows a significant annual difference according to this model. During 2010 the number of cases increased in Bab Zitouna and Bab tété with 73 and 30 cases declared, respectively.

### Distribution of CL and VL by age and sex

**Fig. 9:**
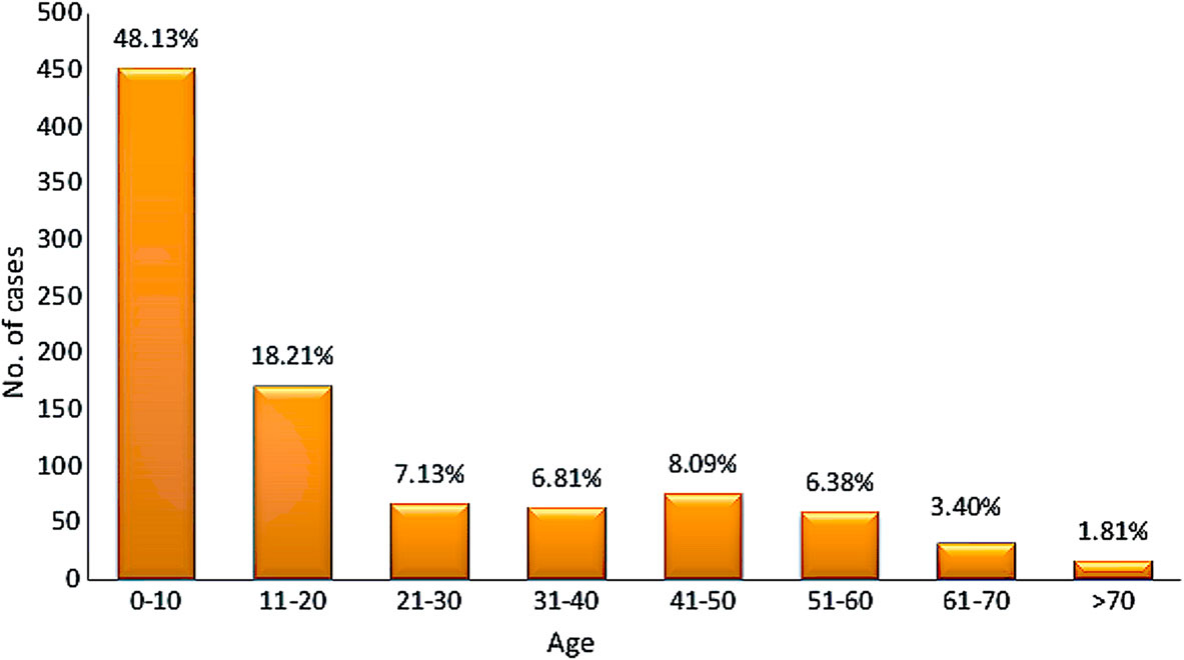
Distribution of leishmaniasis cases in relation to age (2007–2014)

According to the statistical study from all affected sectors of Taza, we found a slight but significant difference in the CL prevalence between genders (Pearson Chi-square test: *χ* ^2^ = 22.6384, *df* = 1, *P* < 0.0001) with predominance of CL in females (57 *vs* 43% for men; sex-ratio F/M = 1.32). Interestingly, no age group was spared from leishmaniasis. Children aged 1 to 10 years lead with 48.13% of identified cases (Pearson Chi-square test: *χ* ^2^ = 1214, *df* = 7, *P* < 0.0001) (Fig. 9). This high percentage could be due to the sensitivity of the skin at this age, weak immune system and the nudity of children, making them more vulnerable to insect bites, and thus most affected by the leishmaniasis.

### Molecular diagnosis

**Fig. 10:**
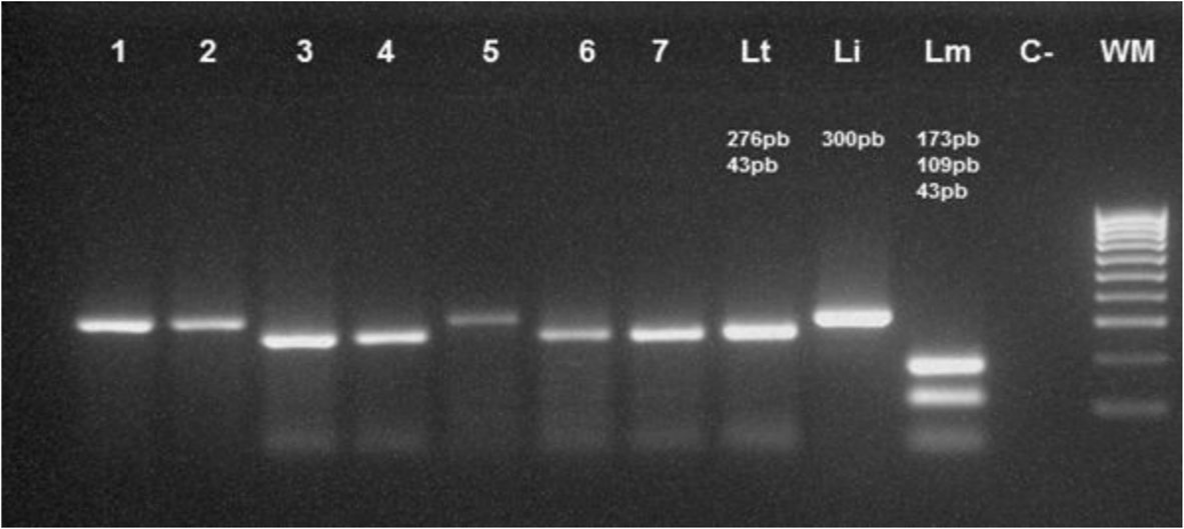
Application of analysis method ITS1-PCR/RFLP on positive slides of *Leishmania* in Taza Province. Lanes 1, 2, 5: *L. infantum*; Lanes 3, 4, 6, 7: *L tropica*; Lane WM: weight marker 100 bp. Positive controls: Lt, *L. tropica*; Li, *L. infantum*; Lm, *L. major*; C-, negative control;

Sixty-three positive slides were analyzed by the molecular study. Amplification of ITS1 confirmed the positive result (s) from direct examination. *Leishmania tropica* and *L. infantum* were the circulating species in Taza Province according to PCR-RFLP analysis with enzyme Mn1-I (Table 1; Fig. 10). PCR-ITS1-RFLP of 3 slides of VL from 3 sectors affected among 4 showed the existence of *L. infantum* (Table 2; Fig. 10).

**Table 1:** Molecular examination results of cutaneous leishmaniasis (CL) slides of affected sectors in Taza Province in 2014

Commune	Sector	Locality	Urban/Rural	Results ITS1	Results RFLP	No. of CL slides
Aknoul	Aknoul	Aknoul	R	Positive	*L. infantum*	1
Oued amlil	Bni frassen	Bnifrassen	R	Positive	*L. infantum*	4
		Douar lakrakra	R	Positive	*L. infantum*	1
	Bouchefaa	Bouchefaa	R	Positive	*L. infantum*	1
		Dour sahla	R	Positive	*L. infantum*	1
	Bouhlou	Aghbal	R	Positive	*L. tropica*	1
		Bnimter	R	Positive	*L. tropica*	2
		Bouhlou	R	Positive	*L. tropica*	8
		Bouhlou	R	Positive	*L. infantum*	2
	Oued amlil center	Boukadous	R	Positive	*L. tropica*	1
		Kaouan	R	Positive	*L. tropica*	1
		Koudia	R	Positive	*L. tropica*	1
		Oued amlil	U	Positive	*L. tropica*	3
		Oued amlil	U	Positive	*L. infantum*	1
Tahla	Matmata	Firdaous	U	Positive	*L. infantum*	1
	Tahla	Hay el fath	U	Positive	*L. tropica*	1
		Tahla	U	Positive	*L. tropica*	3
		Tahla	U	Positive	*L. infantum*	1
		Takaddoum	U	Positive	*L. tropica*	1
Tainaste	Kaf elghar	Kaf elghar	R	Positive	*L. infantum*	1
	Tainaste	Amtaher	R	Positive	*L. infantum*	1
		Oulid	R	Positive	*L. infantum*	1
		Tainaste	R	Positive	*L. tropica*	2
Taza	Taza high	Babmrouj	R	Positive	*L. tropica*	1
		Babmrouj	R	Positive	*L. infantum*	4
		Bab tété	U	Positive	*L. infantum*	2
		Bab tété	U	Positive	*L. tropica*	2
		Babzitouna	U	Positive	*L. tropica*	7
		El yassmin	U	Positive	*L. infantum*	1
		Hay Taoufiq	U	Positive	*L. infantum*	1
		Internat	U	Positive	*L. tropica*	1
		abbatoires	U	Positive	*L. tropica*	1
		Msila	U	Positive	*L. tropica*	1
		Ouledzbair	U	Positive	*L. infantum*	1
		Taza high	U	Positive	*L. infantum*	1

*Abbreviations*: *R*: rural, *U*: urban

**Table 2:** Molecular examination results of visceral leishmaniasis (VL) slides of affected sector in Taza Province in 2014

Commune	Sector	Locality	Urban/Rural	Results ITS1	Results RFLP	No. of VL slides
Taza	Taza high	Bab mrouj	Rural	Positive	*L. infantum*	1
		Bab marzouka	Rural	Positive	*L. infantum*	1
Bni lent	Bni lent	Bni lent	Rural	Positive	*L. infantum*	1

## Discussion

Taza Province is the oldest focus of CL due to *L. tropica* MON-102 [17]. It is known for its crossroad position between several foci of *Leishmania*, namely Taounate, Sefrou and Al Hoceima provinces. This study aimed to update the circulating species of *Leishmania* in Taza Province and to follow the spatio-temporal extension of this disease.

The molecular study of CL in Taza Province has shown for the first time the presence of 41% cases of CL due to *L. infantum* (27/63) together with *L. tropica*. Also, this study shows the coexistence of these two species in the same sectors, namely Taza High, Oued Amlil, Tahla, Tainaste and Bouhlou. The abundance of *L. infantum* among the total could be due firstly to the presence of VL due to *L. infantum* in this province and in the neighboring areas. Secondly, Taza Province belongs to sub-arid and sub-humid areas located in an altitude of 600 m, with the presence of plains and hills but also mountains up to 3250 m. These climatic and geographical conditions are favorable for the existence of *Phlebotomus longicuspis*, *Ph. perniciosus* and *Ph. ariasi* [18]. These sand flies were identified in Taza Province [17] and were largely known for their role in the transmission of CL due to *L. infantum* [19, 20]. These vectors were identified also in Sefrou Province (a neighboring province) [21].

Given that leishmaniasis constitutes a public health problem, it is managed by the National Program against Leishmaniasis. Based on previous work that showed the presence of *L. tropica* MON-102 in Taza Province, the control had access to early diagnosis and treatment, as *L. tropica* was considered anthroponotic. The results of the molecular identification showed the significant presence of *L. infantum*, which changes the strategy of control and surveillance since the dog is the reservoir.

CL due to *L. tropica* has been identified in this province since 1997 [17] and was largely identified in the neighboring provinces in Morocco such as Sefrou [8], Moulay Yacoub [22] and Taounate [23]. It is transmitted throughout *Phlebotomus sergenti*, the proven vector of *L. tropica* in Azilal [24] and Essaouira [25]. This vector was identified in the study area in 1997 [17].

The molecular study of VL has showed the presence of *L. infantum* in the slides examined in Taza Province. These results are in agreement with those found in other studies that prove *L. infantum* is the species responsible of VL in several foci [26, 27]. The zymodeme MON-1 is the most common [28], the second identified zymodeme is MON-24 [11]. Taza Province was known to be a hypoendemic focus of VL (*L. infantum*) until 1995; it is transmitted by *Phlebotomus longicuspis*, and this vector was identified in the study area in 1997 [17].

The transmission cycle of *L. infantum* is zoonotic. In fact, dogs have been implicated as the main reservoir hosts of *L. infantum*. Also, the presence of dogs and their role in the transmission cycle of the disease is regarded as the most important risk factor for leishmaniasis [29]. Therefore, it should be noted that surveillance of the reservoir must be conducted in collaboration with the local collectors, veterinarians, hygiene offices and local authorities. These actions will allow us to monitor the health of human and dog. Thus, an awareness of the population is essential.

In this province, the temporal study shows that the number of cases continues to rise particularly between 2010 and 2014 despite the efforts deployed. This increase can be explained mainly by the active screening conducted following the introduction of a response action plan between 2010 and 2016. Moreover, other factors could cause this increase, i.e. the population growth and movements, and human activities such as landscape modification, which could increase the risk of contracting leishmaniasis by changing of the temperature and humidity of the soil, but also, by causing emigration of infected persons living in rural areas to peri-urban zones where living conditions remain precarious.

A recent study concerning the spatial distribution of CL in Taza Province did not mention the affected sectors. Our geographical study showed that the majority of cases were recorded in three sectors: Taza High (102 cases), Bouhlou (49 cases) and Tahla (38 cases). The other sectors were marked by the appearance of sporadic cases (between one and five). The Taza high sector is the most affected by leishmaniasis especially in the urban localities (Bab Zitouna and Bab tété) with 84%. This sector is very old and located on a mountainous area; it is surrounded by old cracked and unrestored walls with a nearby river and caves that provide daytime resting places for sand flies. The distribution of pathogens and their vectors is related to several factors. In fact, the propagation of leishmaniasis is related to environmental factors such as deforestation, dam construction and irrigation and urbanization systems [1]. Several extension panels are located at the Sebou basin, namely Bouhlou Panel, Taza Aquifer and Ras El Ma Aquifer. Furthermore, to these Hydraulic Resources, the province benefits from the existence of dam Bab Louta with a fill rate of 93 million m^3^. All those explain the high humidity of the region. Moreover, Taza Province is characterized by an average rainfall between 100 and 200 mm/year in arid areas and exceeds 500 mm/year in humid areas. These factors could be favorable for the development of sandflies. Further, Taza Province is among the provinces where the poverty rate is the highest, absolute poverty is over 20% while the established national average is 14.2% [14]. The World Health Organization considers poverty as the most important factor that increases the risk of leishmaniasis; poor living conditions and hygiene can expand the number of breeding sites for sand flies and their access to humans [1].

## Conclusion

The distribution of leishmaniasis in the Province of Taza is linked to several factors such as environmental and socio-economic factors. Moreover, control efforts against this disease must be focused on the Taza High sector (mountain area), considered as an important focus of CL, followed by Bouhlou and Tahla sectors. The molecular diagnosis shows the existence of non-sporadic cases of CL due to *L. infantum* along with *L. tropica*. For better management and control of the disease it is crucial to target the various links of the cycles of the disease which is anthroponotic (*L. tropica*) and zoonotic (*L. infantum* and *L. tropica*).

## Additional file

Additional file 1: Table S1. Distribution of leishmaniasis cases in different localities of Taza Province in relation to year (between 2007–2014) (XLSX 19 kb)

## Abbreviations

CL: Cutaneous leishmaniasis
NIH: National Institute of Hygiene, Morocco
NRLL: National Reference Laboratory of Leishmaniasis, Morocco
VL: Visceral leishmaniasis
